# 3D Virtual Reality Smartphone Training for Chemotherapy Drug Administration by Non-oncology Nurses: A Randomized Controlled Trial

**DOI:** 10.3389/fmed.2022.889125

**Published:** 2022-06-20

**Authors:** Chin-Yun Wang, Chi-Yu Lu, Su-Yueh Yang, Shu-Chun Tsai, Tsai-Wei Huang

**Affiliations:** ^1^Center for Nursing and Healthcare Research in Clinical Practice Application, Wan Fang Hospital, Taipei Medical University, Taipei, Taiwan; ^2^Department of Nursing, Wan Fang Hospital, Taipei Medical University, Taipei, Taiwan; ^3^School of Nursing, College of Nursing, Taipei Medical University, Taipei, Taiwan; ^4^Cochrane Taiwan, Taipei Medical University, Taipei, Taiwan

**Keywords:** chemotherapy, administration, virtual reality, objective structured clinical examination, OSCE

## Abstract

Chemotherapy agents are cytotoxic materials. Thus, there is a need for the operators to be familiar with the knowledge and procedures before operation. We conducted a randomized controlled trial investigating the effectiveness of an immersive 3D VR teaching of chemotherapy administration operated in a smartphone coupled with a visual and audio device. We adopted a two-arm single-blind design and recruited 83 nurses, and they were randomized using a cluster approach. The VR group learned chemotherapy administration through VR, while the controlled group learned through document reading. The Knowledge and Attitude of Chemotherapy Administration (KACA) was administrated before the intervention, while the Objective Structured Clinical Examination (OSCE) and the Checklist of Action Accomplishment (CAA) were administrated one month after the intervention. The VR group scored higher than the controlled group in the CAA (95.69 ± 5.37 vs. 91.98 ± 9.31, *p* = 0.02) and the OSCE (73.07 ± 10.99 vs. 67.44 ±10.65, *p* = 0.02). Stepwise regression demonstrated that service years, an education level of undergraduate or above, and VR exposure contributed positively to the OSCE score (adjusted R^2^ = 0.194, *p* = 0.028). The use of VR improves the learning efficacy of chemotherapy administration in non-oncology nurses. We recommend using VR as a teaching tool for chemotherapy administration and other chemotherapy-related skills in a VR learning group with senior nurses with higher education levels as advisors. The study provides an approach to online training, especially during the COVID-19 pandemic. (CONSORT 2010 guidelines, registry number: NCT 04840732).

## Introduction

Chemotherapy agents are cytotoxic materials, and exposure to the operators might cause acute physical harms such as skin rashes, sore throat, chronic cough, dizziness, headache, eye irritation, hair loss, and allergic (1, 2), and more importantly, increase the risk of cancer (3). The past harm studies of healthcare professionals have reported an extremely high exposure to chemotherapy drugs among medical professionals. In the United States, an estimated 8 million health care workers are potentially exposed to hazardous drugs (4). Thus, chemotherapy drugs must be handled with extreme care. Chemotherapy drug administration is a daily routine procedure in the oncology (5). The oncology nursing staff is usually familiar and skillful with the administration of chemotherapy drugs (6). Such procedures can be challenging for new and inexperienced nurses because it is demonstrated by instructors and not generally practiced by the students themselves in nursing school. Occasionally, nurses of departments other than oncology departments may need to administer chemotherapy drugs, and these nurses may be at a higher risk of exposure and injury because of a lack of adequate knowledge and training.

To be familiar with the chemotherapy administration procedure, traditionally, on-the-job education or training is usually provided to those who will operate the procedure or are likely to operate the procedure. The most common approaches for learning the chemotherapy drug administration knowledge and skills are attending lectures, reading the technical manual or standard operating procedure sheet, and absorbing experiences from the on-site demonstration. In the worst situation, the nursing staff must perform the chemotherapy drug administration right away without a chance to practice. Digitalization of medical orders and procedures has contributed to the correctness of administration procedures, but this does not resolve the problem of effective learning for nurses requiring the techniques.

Simulation-based nursing education is an increasingly popular teaching method. It provides students with the opportunity to practice their clinical and decision-making skills through a variety of experiences. Virtual reality (VR) is defined as an interactive 3D visualization-based communication interface that allows users to interact and integrate with different sensory inputs that simulate important aspects of the real-world experience (7). VR-simulator based training can provide a teaching strategy and act as a facilitator of knowledge retention, clinical reasoning, increased learning satisfaction and self-efficacy (8). Simulations can help virtually practice motor control skills, decision-making skills, and communication skills (9, 10). Simulation methods including low-fidelity to high-fidelity simulations, which may be related to the technical complexity of the desired implementation and the required cost. However, they were not statistically significantly different between studies based on measures of clinical performance (11).

The potential of various VR techniques of different levels and forms has been explored as a teaching approach in medical practice, e.g., sputum suctioning (12). The fidelity of the simulation should be appropriate, even a low-fidelity VR simulator can dramatically improve clinical performance (8). In the current study, we conducted a randomized controlled trial investigating the effectiveness of an immersive three-dimensional (3D) VR teaching of chemotherapy administration operated in a smartphone coupled with a visual and audio device.

## Materials and Methods

**Figure 1:**
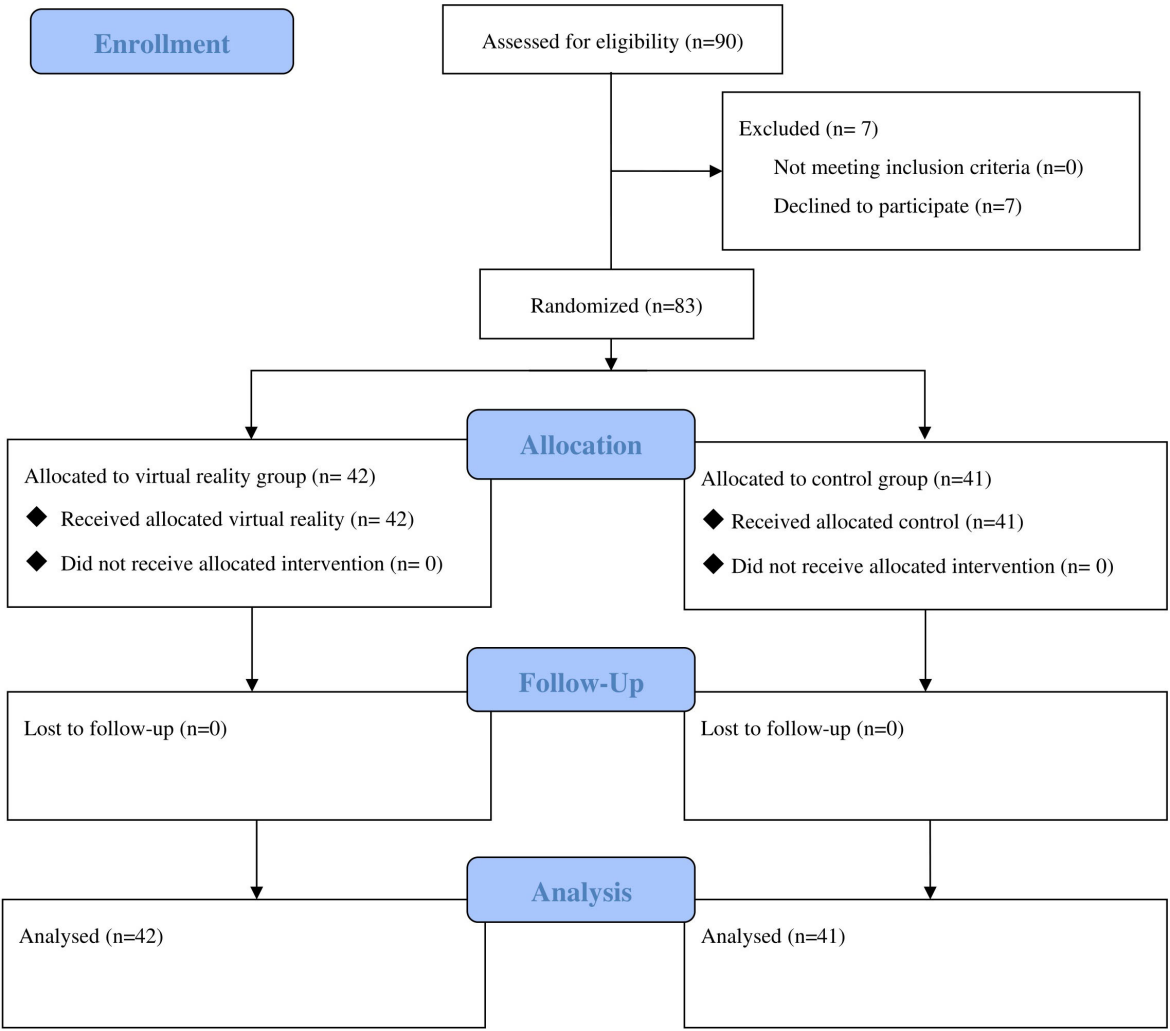
CONSORT flow diagram.

Figure 1 showed the information reported by CONSORT 2010 checklist when reporting a randomized trial (13).

### Sample Size

According to Cohen (14), for an estimated effect size of 0.80, the approximate sample size required is 26 for each group when the power is set at 0.80 and type I error at 0.05 (14). The parameters necessary for the calculation were obtained from Bayram et al., it was determined that the number of individuals required to reject the null hypothesis (12). Therefore, the sample size (*N* = 83) in this study (42 experimental group and 41 control group) was large enough to detect the VR effect.

### Eligibility

Potential participants were nurses who had been employed for at least three months. The inclusion criteria were: (i) a need to operate chemotherapy techniques; (ii) aged at least 20 years old. Subjects were excluded if they were (i) senior oncology nurses or (ii) could not or fail to participate fully in the training program.

### Two-Arm Single-Blind Design

Since non-oncology wards still receive cancer patients who are not hospitalized for cancer problems, nurses still need to follow doctor's orders when chemotherapy is needed. Or it is one of the training contents when the nursing staff of different departments are alternately trained. There were eight non-oncology wards in our hospital with 90 nurses. The Institutional Review Board of Taipei Medical University, Taiwan approved this study (TMU-JIRB, N201801073). Among them seven declined to join the study and 83 nurses were recruited in the study. We performed a two-arm single-blind, parallel randomized controlled trial. On agreeing and signing informed consent, the nurses were randomized using cluster approach, i.e., computer randomization (excel RANDBETWEEN) by nursing station. Thus, nurses of an allocated nursing station would all be either VR or controlled participants. To prevent the two groups of participants from sharing experimental content with each other, the control group first obtained educational documents (standard group). The education materials for the VR group were obtained at the nursing station. After the completion of OSCE, VR teaching materials were also provided to the control group. Both data collectors and outcome adjudicators were blinded. The data analyst receives the data and analyzes it after the experiment. The trial registration number is clinicaltrials.gov. NCT 04840732.

### Objective Structured Clinical Examination

During an Objective Structured Clinical Examination (OSCE), students address and resolve various simulated clinical situations representing real clinical practices (15). The examination has been recognized as an effective assessment tool for evaluating students' clinical performance (16–18). OSCE may be a powerful tool in the evaluation of clinical competence in nursing and that it may also be an effective facilitator for learning to perform clinical skills in nursing (19). The design of the clinical scenario simulation is based on standard hospital chemotherapy administration and guideline. Standardized cases are expected to be treated with chemotherapy. The staff must checking orders, preparing chemotherapy drugs, checking chemotherapy drugs, perform chemotherapy administration, and chemotherapy waste sorting and disposal, with a total score of 100 points. The OSCE assessment was divided into three stations, each of which was assessed by a senior oncology nurse. The three assessors do not know which group the participants belonged to.

### Intervention and Control Group

The learning objectives were to operate the chemotherapy administration process and chemical waste disposal procedures correctly and safely. The training consists of five parts: checking orders, prepare the premedication for chemotherapy, check chemotherapy drugs, perform chemotherapy administration, and chemotherapy waste sorting and disposal for both VR intervention and control groups. All participants were informed that an OSCE for chemotherapy drug administration was coming after 1 month.

**Figure 2:**
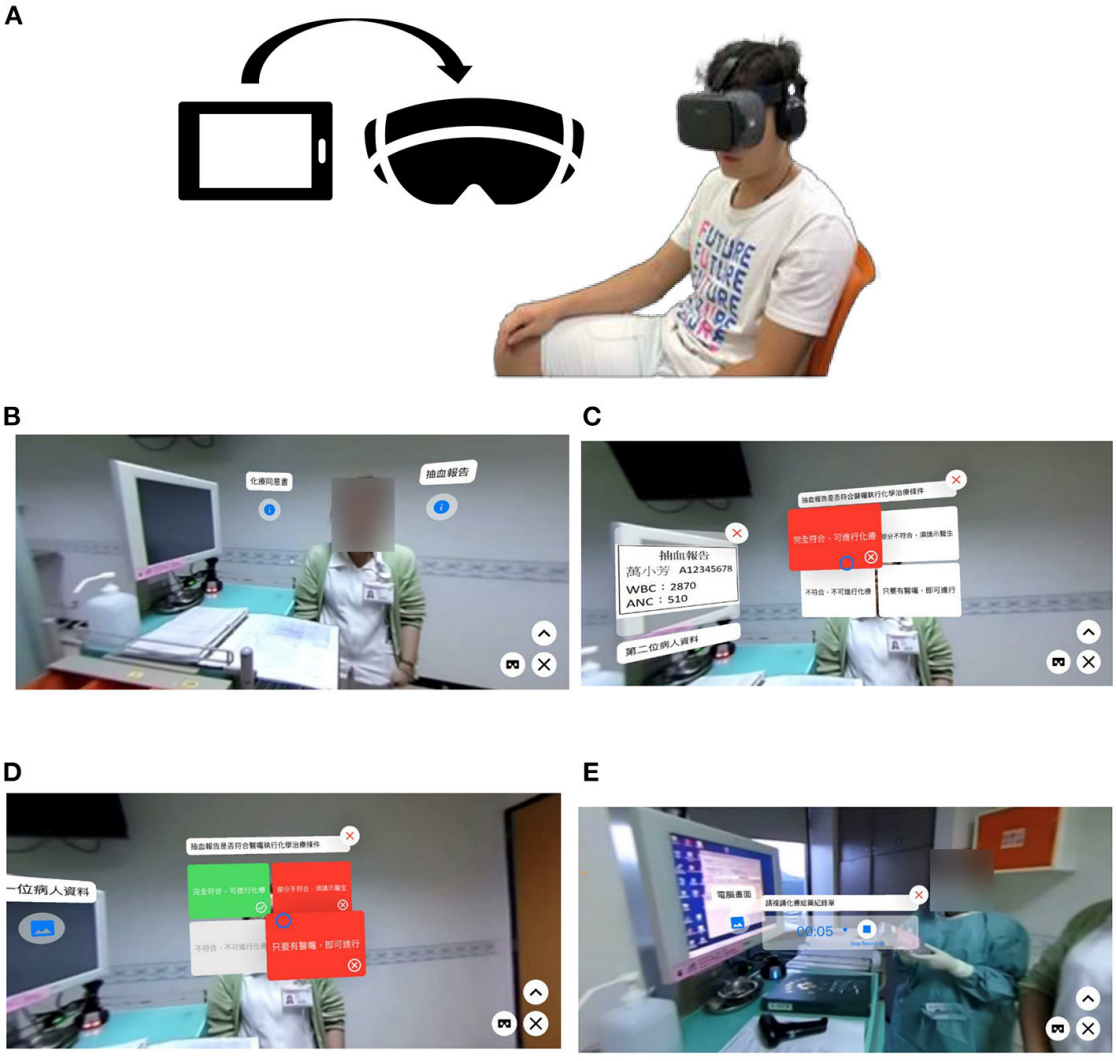
Educational content and environment settings for the VR group. **(A)** Smartphone and VR goggles; **(B)** Before chemotherapy, you can use the select button to view the contents of the documents that need to be confirmed; **(C)** Check the blood draw report to see if chemotherapy can be performed. If the wrong selection is made, the next step cannot be performed; **(D)** If the correct answer is selected, proceed to the next step; **(E)** To practice and to record the double check medication process.

To mask the allocation, we arranged the controlled participants to be exposed to the target education materials before the intervention group. The educational materials for the VR group are filmed and produced using EduVenture VR (Centre for Learning Sciences and Technologies, The Chinese University of Hong Kong, Shatin, Hong Kong) and 360 VR camera. This information can be used by downloading the APP to the public smartphones of the nursing station. Each nursing station is equipped with VR glasses for viewing VR through a smartphone. Participants can take the VR session at the nursing station or take it home. The App can include simple interactions, including multiple-choice, true-false and recording question. Figure 2 presents the VR groups using smartphones and shows the approach to the interaction problem. For example, the number of white blood cells in the blood, such as ANC (absolute neutrophil count), needs to be confirmed before chemotherapy. Participants need to answer the selected item correctly and they can move on to the next step. In addition, the recording function can be used for double check of the administration dosing process.

In the VR group, the participants used a smartphone paired with VR glasses and audio device, visually experienced the process from initial preparation of the drug to final disposal with chemical waste using their smartphones. They were instructed to learn the chemotherapy administration skills from the software or APP for 1 month at least once a day. When the VR software was run for the first time, the experimental group operated with the assistance of the investigator to confirm that it was running normally.

In the control group, the participants received standard operating procedures for chemotherapy administration and were instructed to learn from the document at least once a day for a month.

Both groups were reminded weekly by investigator using the LINE messaging software to read documents or watch VR app. Voluntary learning by two groups of nursing staff.

### Pre-test and Post-test

Participants were asked to do a pre-test 2 or 3 days before the initiation of the trial. A post-test about knowledge and attitude of chemotherapy and the effectiveness of two educational approaches was conducted 1 month after the trial. The post-test included self-assessment and OSCE for chemotherapy administration procedures. The assessors for OSCE were senior oncology nurses blinded to allocations. After the examination, the assessors discussed the process during the OSCE and recorded the common problems observed.

### Measurements and Outcomes

The Knowledge and Attitude of Chemotherapy Administration (KACA) was a questionnaire designed by our research group measured in both the intervention group and control group at baseline. A survey using the Checklist of Action Accomplishment after intervention (CAA), another questionnaire designed by us, and the Objective Structured Clinical Examination (OSCE), were conducted 1 month after the intervention. We designed ten questions for KACA based on previous literature (4, 20–22). The expert validity of KACA was 0.82, and the Cronbach α was 0.9. The score of each question was measured using the Likert scale, with 1 as totally disagree and 5 as totally agreed. A higher score implies positivity toward the knowledge and attitude of chemotherapy drug administration.

We listed eight check points to be inspected in the checklist of action accomplishment (CAA) with a total score of 100. The expert validity of the checklist was 0.88, and the Cronbach α was 0.91. The OSCE for this study used the same eight check points to be inspected. The participants' KACA and CAA were self-reported, and two senior nurses scored the OSCE. The internal validity of the OSCE was 0.9.

### Statistical Analysis

The Chi-square tests and independent *t*-tests were used to examine the differences of categorical and continuous variables between two groups for demographic variables, while the independent *t*-tests were used to examine the outcome of interest (KACA, CAA, and OSCE). We also examined the potential effects of demographic variables on OSCE using *t*-test and analysis of variance (ANOVA) where appropriate. Stepwise multivariate regression was used to test the contribution of predictability of potential variables on OSCE. SPSS Statistics 24 was used for the statistical analyses.

## Results

### General Characteristics

All participants were young adults with several service years in the hospital. The number of participants of education levels of undergraduate and above was slightly higher than junior college in the sample. Most of the participants had have a registered nurse level (*N*) 1 or 2, and about half of them had ever conducted or assisted chemotherapy drug administration in the past month. No significant differences were found for the above variables (Table 1).

**Table 1:** General characteristics of the study participants (*N* = 83).

**Characteristics**	**Virtual reality** **(*n =* 42)**	**Control** **(*n =* 41)**	***t*-value**	***p*-value**
	**M ±SD**	**M ±SD**		
Age	28.31 ± 5.76	26.78 ± 4.35	−1.36	0.18
Service years	5.79 ± 5.22	4.51 ± 4.10	−1.24	0.22
	***n*** **(%)**	***n*** **(%)**	**χ^2^ value**	***p*** **-value**
Education			0.02	0.90
Junior college	19 (45.2%)	18 (43.9%)		
Undergraduate and above	23 (54.8%)	23 (56.1%)		
Registered nurse level (N)			1.17	0.56
N0	5 (11.9%)	5 (12.2%)		
N1	10 (23.8%)	14 (34.1%)		
N2 and above	27 (64.3%)	22 (53.7%)		
Ever conducted or assisted chemotherapy drug administration in the past month			0.11	0.75
No	19(45.2%)	20(48.8%)		
Yes	23(54.8%)	21(51.2%)		

*M, mean; SD, standard deviation*.

### Outcomes

**Figure 3:**
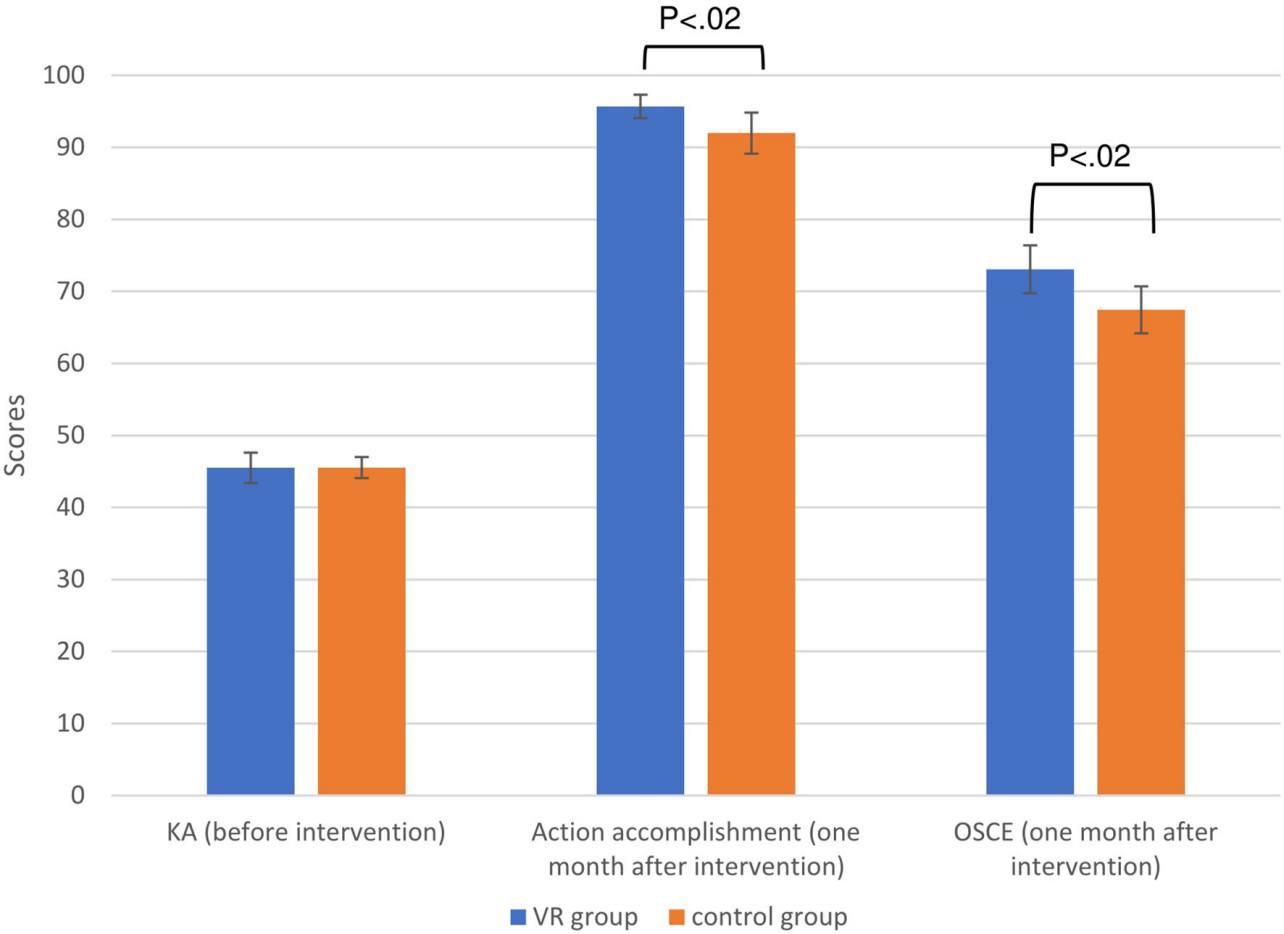
Knowledge and Attitude of Chemotherapy Administration before intervention (maximum score 50), Checklist of Action Accomplishment after intervention (maximum score 100), and OSCE score (maximum score 100) after intervention among the participants (*N* = 83). Error bars = 95 % confidence interval. KA, Knowledge and Attitude; OSCE, Objective Structured Clinical Examination score; VR, virtual reality.

Figure 3 summarizes the results of KACA before intervention, and CAA and OSCE score 1 month after the intervention. No difference was found between VR and control groups (45.50 ± 6.99 vs. 45.54 ± 4.78, *p* = 0.98) in the KACA. However, the CAA score of the VR group was significantly higher than that of the control group (95.69 ± 5.37 vs. 91.98 ± 9.31, *p* = 0.02). Similarly, The OSCE score of the VR group was significantly higher than that of the control group (73.07 ± 10.99 vs. 67.44 ±10.65, *p* = 0.02). On a 60-point pass criterion, OSCE failure rates were 9.5% in the VR group and 19.5% in the control group.

### Contents of CAA and OSCE

We summarize the statistical outcomes of contents of the CAA and OSCE in Supplementary Table 1. None of the contents in CAA were significantly different between the two groups except item 8-8 in which the VR group demonstrated a higher score than the control group (3.83 ± 0.44 vs. 3.41 ± 1.07, *p* = 0.024). For OSCE, the VR group demonstrated a significantly higher score in item 3 (i.e., carrying out required premedication or pretreatments before chemotherapy administration) compared with the control group (3.05 ± 1.72 vs. 1.76 ± 2.01, *p* = 0.002). The second item that showed a significant difference between the two groups was item 7 (i.e., transfer the chemotherapy drug to be administrated and the clinical record sheet by two nursing staff to the patient unit); the VR group demonstrated a significantly higher score in item 7 compared with the control group (3.43 ± 1.42 vs. 2.63 ± 1.92, *p* = 0.036). Also, the VR group showed a higher score in item 8-8 (ending procedures after finishing chemotherapy drug infusion before leaving patient's bed) (3.76 ± 0.79 vs. 3.12 ± 1.35, *p* = 0.011). The VR group demonstrated a non-significant higher score in Item 6-2 (i.e., a box containing the chemotherapy drug to be placed on the cart layered with absorbent waterproof paper) compared with the control group (3.90 ± 0.62 vs. 3.51 ± 1.33, *p* = 0.090), so as the item 8-7 (checking infusion parameters, sign, and record after fixing chemotherapy infusion drugs) (4.29 ± 2.80 vs. 3.22 ± 2.42, *p* = 0.067). However, the VR group demonstrated a non-significant lower score in the VR group compared with the control group (3.43 ± 1.42 vs. 3.90 ± 0.62, *p* = 0.053). The other 11 variables did not show a difference between the two groups.

### Effects of Demographic Variables in OSCE

Table 2 summarizes the results of the t-test and ANOVA examining the effects of demographic variables in OSCE. Nursing staff with an undergraduate education level had a significantly higher OSCE score than those with a junior college education level (73.4 ± 9.2 vs. 66.4 ± 12.1, *p* = 0.003). The OSCE score was also positively correlated with their service years (*r* = 0.345, *p* = 0.001). No difference was observed between those who ever conducted or assisted chemotherapy drug administration in the past month than those who did not (70.9 ± 9.7 vs. 69.5± 12.6, *p* = 0.57). A non-significant trend toward increment of OSCE was observed with the higher registered nurse levels (*p* = 0.068).

**Table 2:** OSCE score between VR and control groups (*N* = 83) in demographic variables and experience of chemotherapy administration.

**Characteristics**	**Objective Structured Clinical Examination score**		
	**Pearson's correlation**		**p-value**
Service years	0.345		0.001
	**M** **±SD**	**t-score**	**p value**
Education		−3.01	0.003
Junior college	66.4 ± 12.1		
Undergraduate or above	73.4 ± 9.2		
Ever conducted or assisted chemotherapy drug administration in the past month		−0.58	0.57
No	69.5 ± 12.6		
Yes	70.9 ± 9.7		
	**M** **±SD**	***F*** **-score**	***p*** **-value**
*N*-level		2.78	0.068
N0	66.5 ± 11.2		
N1	67.1 ± 9.5		
N2 and above	72.6 ± 11.4		

*M, mean; SD, standard deviation; OSCE, Objective Structured Clinical Examination score; VR, virtual reality*.

### Regression

Table 3 summarizes the results of stepwise regression of all variables in Table 2 against OSCE score. The past month's experience of chemotherapy and the registered nurse level were excluded from the model. The three variables that remained in the model were service years, an education level of undergraduate or above, and being a participant in the VR group, all of which contributed positively to the OSCE score (adjusted *R*^2^ = 0.194, *p* = 0.028).

**Table 3:** Stepwise regression of the variables affecting OSCE score (*N* = 83).

**Variables**	**β**	**SE**	**Adjusted *R*^2^**	***p*-value**
Service years	0.58	0.25	0.108	0.019
Undergraduate or above (Ref.: Junior college)	5.70	2.29	0.154	0.015
Virtual reality group (Ref.: Control)	4.96	2.22	0.194	0.028

*Objective Structured Clinical Examination score = 55.91 + 0.58 × service years + 5.7 × education + 4.96 × group*.

## Discussion

Our study found that the VR group had a higher score in CAAAI and OSCE compared with the control group. Regression model against showed that service years, higher education (undergraduate or above), and use of VR contributed a higher OSCE score. The significantly higher score in OSCE among VR users supports that the better learning efficacy is not a Horthorne Effect simply because of participation.

Overall speaking, although the results of CAA and OSCE simultaneously showed that VR helps to learn chemotherapy drug administration, detailed analysis of the contents of learning items showed that non-oncology nurse had different views of what is known or understood in comparison with the objective OSCE. Interestingly, the only item that showed the same difference between the CAA and OSCE scores was item 8-8, the count-backward second checkpoint of the procedures. One possible explanation is that the learners were losing their alertness because the procedures are coming to an end, and VR provides a complimentary approach to save the missing steps (23).

Psychomotor ability refers to a wide range of actions involving physical movement related to conscious cognitive processing. This concept has been extended to medical teaching that presumes the acquisition of technical skills are stemmed on the formation of a cognitive entity by repetitive training. Learning psychomotor skills involves conveying theoretical knowledge to physical actions (24). The learning of psychomotor skills is even more important to nursing students or staff because practice and direct usefulness are the major goals of the nursing profession. VR represents one of the modern technology that allows repetitive training to support conveying the abstract knowledge to physical actions at any time and place (25), thus fulfilling the psychomotor ability learning theory. VR provides a scenario linking physical movements to conscious cognitive processing to impress users. The newly built skills will be activated and quickly adapted and adjusted when the real scenario appears.

The regression of the variables affecting OSCE score indicated that service years and higher education enhanced the learning performance of the participants besides the use of VR. These findings are not surprising, as a better foundation probably supports a better learning efficacy. On the other hand, the results also imply that VR skills could be helpful for practicing nurses. In many cases, medical professionals have to be involved in quick-changing operations, like that encountered in Covid-19 vaccination and treatment (26). Our study supports the plausibility of VR in terms of time, cost, and risk during emergent training. Our VR simulators are even low-fidelity devices, yet still enhance training. Expertise comes from deliberate practice, and low-fidelity VR simulators give users more opportunities to practice (11). Currently, our hospital is still using the traditional teaching method for teaching chemotherapy drug administration in non-oncology nurses. There are a few potential problems when promoting the use of VR. First, the corrections or amendments of VR teaching contents require re-filming or editing, which require additional manpower, material resources and costs. Secondly, some nurses tend to have a quick look at the key points rather than the whole procedures in the actual scenario, and this behavior appears inconvenient when operating the VR. Even so, with the advancement of technology, it is believed that these problems will be solved in the future.

The Covid-19 pandemic, which is still an ongoing issue worldwide, has posed a problem for the learners to practice in a relatively dangerous environment. The teachers or instructors must focus on their real clinical work more while supervising the learners who are not or less familiar with the target situations. Some preliminary teaching schemes have been abruptly and hastily put on stage, e.g., online case discussions with patient details without a patient or acting patient, which can be impractical as the teaching purposes are to practice. A simulation-based training provides a close-to-real tailored implementation strategy in this revolutionary period (26). Because service years and higher education were two contributing factors to the OSCE besides VR exposure, formation of VR learning group with senior nurses with higher education levels as advisors might enhance the actual learning efficacy.

There are a some limitations in this study. First, our sample size is relatively small and thus the statistical power. Second, the current restriction of mobile models' central processing unit and storage size reduces users' intentions to download the App. Nevertheless, this issue will be resolved sooner or later with the progress of mobile technology. Third, no compliance reporting was done despite the study design planning participants to conduct the trial at least once a day for a month. Fourth, there are some technical differences between different departments when operating the chemotherapy drug administration, e.g., patients of the gynecological department typically require additional infusion in the abdominal cavity. In contrast, for the children with leukemia of the pediatric department, no specific infusion is used. That means different sets of VR are required. Finally, the current study did not involve debriefing. Debriefing is essential in classic learning as well as in simulated learning. Indeed, the means for evaluating simulation debriefing are lacking. Additional efforts should be placed on this issue in the future.

## Conclusion

VR is becoming one of the potential teaching tools in medicine in the era of high-speed progression of computer and digital technology. It is easy to use and does not require abundant training to fit in the teaching materials. Our study indicates that the use of VR improves the learning efficacy of chemotherapy drug administration at least partially in non-oncology nurses. We recommend using VR as a teaching tool for chemotherapy drug administration and other chemotherapy-related skills in a VR learning group with senior nurses with higher education levels as advisors. With the advances of VR technology, more packaged functions, e.g., interactive function, are probably available in the shortcoming future. Also, the importance of human-designed VR teaching is prominent during the period of virus pandemics.

## Data Availability Statement

The original contributions presented in the study are included in the article/Supplementary Material, further inquiries can be directed to the corresponding author/s.

## Ethics Statement

The studies involving human participants were reviewed and approved by Institutional Review Board of Taipei Medical University, Taiwan approved this study (TMU-JIRB, N201801073). The patients/participants provided their written informed consent to participate in this study.

## Author Contributions

C-YW and C-YL are nurse leaders and educator in oncology nursing department. They contributed to the conception of the study, the acquisition, analysis and interpretation of data. S-YY and S-CT are nurse head in internal medicine nursing department and contributed to the acquisition of data, collect the data and approving the current version submitted for publication. T-WH is an Associate Professor of School of Nursing in Taipei Medical University and contributed to the conception of the study, the analysis and interpretation of data, revising the manuscript and approving the current version submitted for publication. All authors contributed to the article and approved the submitted version.

## Funding

This study was funded by Wan Fang Hospital of Taiwan (Grant Number: 107-wf-eva-34).

## Conflict of Interest

The authors declare that the research was conducted in the absence of any commercial or financial relationships that could be construed as a potential conflict of interest.

## Publisher's Note

All claims expressed in this article are solely those of the authors and do not necessarily represent those of their affiliated organizations, or those of the publisher, the editors and the reviewers. Any product that may be evaluated in this article, or claim that may be made by its manufacturer, is not guaranteed or endorsed by the publisher.
