# Supplementary material for: 3D Virtual Reality Smartphone Training for Chemotherapy Drug Administration by Non-oncology Nurses: A Randomized Controlled Trial
Source: Front Med (Lausanne). 2022 Jun 20;9:889125. doi: 10.3389/fmed.2022.889125 (PMC9251548; doi:10.3389/fmed.2022.889125)
Supplement: Supplementary file 1 [file Table_1.docx]

Supplementary 1. Self and OSCE-evaluation scores of VR handouts for chemotherapy administration

| Item | Self-evaluation scores | | | Objective Structured Clinical Examination -evaluation scores | | |
| --- | --- | --- | --- | --- | --- | --- |
|  | Mean | SD | *p value* | Mean | SD | *p value* |
| 1. Hand sanitization |  |  |  |  |  |  |
| Control | 3.88 | 0.40 | 0.148 | 2.54 | 1.95 | 0.315 |
| virtual reality | 3.98 | 0.15 |  | 2.10 | 2.02 |  |
| 2. Verifications: patient identification, explanation of the purpose of drug administration and procedure, and confirm smooth injection of the drug (e.g., eye-confirming of blood backflow). |  |  |  |  |  |  |
| Control | 3.73 | 0.63 | 0.184 | 2.54 | 1.95 | 0.442 |
| virtual reality | 3.88 | 0.33 |  | 2.86 | 1.83 |  |
| 3. Carrying out required premedication or pretreatments before chemotherapy administration |  |  |  |  |  |  |
| Control | 3.80 | 0.56 | 0.203 | 1.76 | 2.01 | 0.002 |
| virtual reality | 3.93 | 0.26 |  | 3.05 | 1.72 |  |
| 4. Hand sanitization again before chemotherapy administration |  |  |  |  |  |  |
| Control | 3.66 | 0.66 | 0.823 | 0.98 | 1.74 | 0.182 |
| virtual reality | 3.69 | 0.64 |  | 1.52 | 1.97 |  |
| 5. Correct use of personal safety and protection wearing and devices |  |  |  |  |  |  |
| Control | 3.80 | 0.51 | 0.611 | 3.44 | 1.12 | 0.131 |
| virtual reality | 3.86 | 0.42 |  | 2.95 | 1.72 |  |
| 6-1. Clinical cart: emergency package for hazardous drug leakage or splash to be located on the lowest shelf. |  |  |  |  |  |  |
| Control | 3.29 | 0.96 | 0.070 | 2.15 | 2.02 | 0.154 |
| virtual reality | 3.62 | 0.62 |  | 2.76 | 1.87 |  |
| 6-2. Box containing the chemotherapy drug to be placed on the cart layered with absorbent waterproof paper. |  |  |  |  |  |  |
| Control | 3.71 | 0.78 | 0.195 | 3.51 | 1.33 | 0.090 |
| virtual reality | 3.88 | 0.33 |  | 3.90 | 0.62 |  |
| 7. Transfer the chemotherapy drug to be administrated and the clinical record sheet by two nursing staff to the patient unit. |  |  |  |  |  |  |
| Control | 3.80 | 0.56 | 0.064 | 2.63 | 1.92 | 0.036 |
| virtual reality | 3.98 | 0.15 |  | 3.43 | 1.42 |  |
| 8-1. Nursing staff protected with complete safety dress and wearing put the absorbent waterproof paper on the operating desk (an adjustable sliding plate), and take out the chemotherapy drug to be administrated and confirm the completeness and intact of the drug. |  |  |  |  |  |  |
| Control | 3.68 | 0.61 | 0.220 | 3.32 | 1.52 | 0.509 |
| virtual reality | 3.83 | 0.49 |  | 3.52 | 1.31 |  |
| 8-2. Patient identification (scan patient’s name band and drug’s label) |  |  |  |  |  |  |
| Control | 3.95 | 0.31 | 0.323 | 3.90 | 0.62 | 0.053 |
| virtual reality | 4.00 | 0.001 |  | 3.43 | 1.42 |  |
| 8-3. Confirmation of information on clinical record sheet and computer including drug name, infusion solution, dosage, route, and time by two registered nursing staff. |  |  |  |  |  |  |
| Control | 6.17 | 1.41 | 0.063 | 4.46 | 3.32 | 0.907 |
| virtual reality | 6.64 | 0.76 |  | 4.55 | 3.20 |  |
| 8-4. Calculation of drug dosage and infusion speed (mL/h), record the speed on the clinical record sheet above the signature column of the sheet using a black ball pen. |  |  |  |  |  |  |
| Control | 5.76 | 1.48 | 0.172 | 2.10 | 2.91 | 0.580 |
| virtual reality | 6.17 | 1.23 |  | 1.79 | 2.14 |  |
| 8-5. Reconfirmation of the smooth flow of the venous route before chemotherapy drug administration, and the connection is firm and correct. |  |  |  |  |  |  |
| Control | 3.78 | 0.57 | 0.244 | 2.44 | 1.98 | 0.139 |
| virtual reality | 3.90 | 0.37 |  | 3.05 | 1.72 |  |
| 8-6. Chemotherapy agents and infusion bottle to be allocated on the absorbent waterproof paper, the infusion kit to be infixed to the soft bag placed horizontally on the table, and the hard bottle to be infixed vertically on the table. |  |  |  |  |  |  |
| Control | 6.39 | 0.92 | 0.076 | 1.88 | 3.14 | 0.105 |
| virtual reality | 6.71 | 0.71 |  | 3.07 | 3.47 |  |
| 8-7. After fixing the chemotherapy infusion drugs in place, an ungloved nurse set the infusion parameter (volume/time unit) with double confirmation, and sign both names of the signature column if the chemotherapy drug administration clinical record sheet. |  |  |  |  |  |  |
| Control | 6.41 | 0.89 | 0.161 | 3.22 | 2.42 | 0.067 |
| virtual reality | 6.67 | 0.72 |  | 4.29 | 2.80 |  |
| 8-8. After finishing chemotherapy drug infusion, the two operating nursing staff pushes the clinical cart to the patient’s unit; the softbag (or glass bottle) and others are unloaded by the one with safety dress and devices put on; the other nursing staff who is not wearing gloves will then switch to the information system page, scan patient’s and drug’s barcodes to mark the accomplishment of the infusion procedures. |  |  |  |  |  |  |
| Control | 3.41 | 1.07 | 0.024 | 3.12 | 1.35 | 0.011 |
| virtual reality | 3.83 | 0.44 |  | 3.76 | 0.79 |  |
| 8-9. The ungloved nursing staff pushes the clinical card back to the treatment room at the nursing station, clear-up and sort-out, and wash hands. |  |  |  |  |  |  |
| Control | 3.80 | 0.56 | 0.064 | 3.46 | 1.34 | 0.224 |
| virtual reality | 3.98 | 0.15 |  | 3.05 | 1.72 |  |

Abbreviations: M: mean; SD: standard deviation.
